# Non-causal Explanations in the Humanities: Some Examples

**DOI:** 10.1007/s10699-023-09910-3

**Published:** 2023-04-06

**Authors:** Roland den Boef, René van Woudenberg

**Affiliations:** Free University: Vrije Universiteit Amsterdam, Amsterdam, The Netherlands

**Keywords:** Non-causal explanations, Humanities, History, Linguistics, Archaeology, Literary studies, Explanatory pluralism

## Abstract

The humanistic disciplines aim to offer explanations of a wide variety of phenomena. Philosophical theories of explanation have focused mostly on explanations in the natural sciences; a much discussed theory of explanation is the causal theory of explanation. Recently it has come to be recognized that the sciences sometimes offer respectable explanations that are non-causal. This paper broadens the discussion by discussing explanations that are offered in the fields of history, linguistics, literary theory, and archaeology that do not seem to fit the causal theory of explanation. We conducted an exploratory survey in acclaimed humanities textbooks to find explicitly so-called explanations and analyze their nature. The survey suggests that non-causal explanations are an integral part of the humanities and that they are of distinct kinds. This paper describes three kinds that are suggested by our survey: teleological, formal, and normative explanations. We suggest that such humanistic explanations strengthen the case for explanatory pluralism.

## Introduction

Over the past fifty years, many theories of explanation have focused on causal explanations as they are offered in the natural sciences; these explanations draw attention to what in the world is in part at least causally responsible for what we want to explain (Lewis, 1986; Salmon, 1984; Woodward, 2003). At the same time, many noted that the sciences are also the home of explanations of other kinds. There are explanation by identification—the explanation of thermal phenomena consisted in the discovery that temperature *is* mean molecular motion (Achinstein, 1983). There are also explanations through unification—Newton was able to explain a wide array of phenomena by unifying them through his three laws of motion together with his universal law of gravitation: Kepler’s three laws of planetary motion, the tides, the motion of comets, projectile motion, and pendulums (Kitcher, 1981). And more recently, attention has been given to yet other kinds of non-causal explanations, *formal* explanations (Lange, 2016; Reutlinger & Saatsi, 2018). For example, there is a formal, mathematical explanation of the fact that a mother fails to evenly divide 23 strawberries among her four children (Lange, 2016, p. 6). Also, the disposition of a ball to roll down inclined planes has been explained by reference to the disposition’s being *grounded* in the ball’s being spherical (Audi, 2012, p. 687). In this paper, we focus on alleged explanations in the humanities, and we argue that among them are also explanations of a non-causal variety. We discuss what practitioners of the humanities call ‘explanations’ and have selected examples from history, linguistics, archaeology, and literary theory.

What motivates our discussion is our wish to get a better understanding of the explanatory efforts in the humanities. Humanistic explanations, as we will call them, are underrepresented in the philosophy of explanation. True, in the English speaking world the sciences and humanities are sometimes conceived as worlds apart. In the continental tradition, however, the humanities are held to be a form of *Wissenschaft*, along with the natural, social and biomedical sciences. Given that the humanities also produce high-grade knowledge, including what its practitioners call ‘explanations’, this seems reasonable enough. And we shouldn’t forget that there is a venerable precedent for going back to a broader view of explanation, one that explicitly includes the humanities, namely the work of Carl Hempel (Hempel, 1942, 1965; Hempel & Oppenheim, 1948). Hempel’s first paper on explanation bore the title “The Function of General Laws in History” (Hempel, 1942). Although the Covering Law model proved to unsatisfactory, it is quite possible to make progress in our thinking about humanistic explanations without committing ourselves to Hempel’s explanatory monism, i.e. his insistence that explanations need to refer to general laws.

This paper is an exploratory foray into various kinds of non-causal explanation that can be found in the humanities. It is exploratory and hence limited in five respects. (1) We don’t offer an exhaustive overview of *all* the kinds of putative non-causal explanations we can find in the humanities; we discuss only three such kinds. (2) We *only* suggest that these kinds of explanations are non-causal, and point to typical features of them, but we don’t offer full-blown accounts of each kind. (3) We don’t discuss the interrelations between the three kinds of putative explanations. (4) We don’t offer estimations of the frequency with which these kinds of putative explanation are offered. (5) We are not going to discuss the question of whether some of these putative kinds of explanations could, perhaps, be reduced to causal explanations.

This paper is organized as follows. Section 2 offers some initial clarification of key terms: ‘explanation’, ‘causal explanation’ and ‘humanities.’ Sect. 3 describes our method of selecting the three kinds of explanation we shall discuss. In the subsequent sections, we discuss intentional explanations (Sect. 4), formal explanations (Sect. 5), and normative explanations (Sect. 6). We end with stating our conclusions (Sect. 7).

## Preliminaries: Explanation, Causal Explanation, and the Humanities

First, what is an explanation? This is a vexed question! Newton-Smith goes so far as to say that it is a sort of scandal in the philosophy of science that “we are a very long way from having [a] single unifying theory of explanation” (Newton-Smith 2002, p. 132). Instead, what we have, as indicated above, are explanations by reference to causation, to identities, to unification, to formal properties, and to other things as well, without deeper insight into what it is that makes each of these forms of explanation explanatory. This raises the problem of how to pick out, in the sciences as well as in the humanities, genuine explanations and distinguish them from spurious ones?

For the purposes of this paper, we cope with this problem in a way that is inspired by Roderick Chisholm’s discussion of the problem of the criterion (Chisholm, 1982: 61–75). This means that, given the aim of identifying explanations in the humanities, we pay attention to two things that potentially can be at odds with each other. On the one hand, we pay attention to what practitioners of the humanities *call* ‘explanations’. On the other hand, we pay attention to general intuitions about what an ‘explanation’ *is*. So we pay attention to proffered *examples* or instances of explanations in the humanities, as well as to presumed *criteria* for something’s being an explanation. And potentially these can be in tension with each other. Before we can see *how* that is possible, we should first state some widely shared and basic intuitions about what an explanation *is*, i.e. intuitions about what the criteria for something’s *being* an explanation *are*.

To begin with, there is the widely shared intuition that an explanation is something that provides understanding, renders something intelligible (Newton-Smith 2002: 131). And there are various ways in which something can be rendered intelligible. One way is this: there is a ‘system’ with various ‘parts’ that interact; to understand that system is to see *how* the parts interact, i.e. how the workings of certain parts influence the workings of other parts. To understand the solar system, or the elections in Greece is to see how the parts of the solar system hang together, how the relevant laws, institutions, electorate and officials in Greece interact. Another way is this: to understand something is to see its possibilities and impossibilities; to use Stephen Grimm’s felicitous phrase, to understand a phenomenon is to ‘peek into the modal space surrounding’ it (Grimm, 2016, 2019). To understand the solar system, or the elections in Greece is to see what is possible and impossible for the solar system (impossible that the planets rotate around the sun in inversedirections), and for the Greek elections (impossible that a citizen will be crowned king). What would seem to be the same intuition has also been formulated in terms of counterfactual dependence (Reutlinger, 2016): to explain X by reference to Y, is to point out that X counterfactually depends on Y, meaning that if Y were other than it is, X would be otherwise too. Yet another way is this: to understand something is to see that, given certain other things, it was to be expected that it would happen. Given many things that we know, it was to be expected that the planets don’t orbit the sun in fully regular fashion, and also that only persons with Greek nationality have voting rights in Greece.

Another widespread intuition about explanation is that it is an answer to a why-question (Van Fraassen, 1980, Ch. 5; Salmon, 1992). If you can answer the question why the moon always shows the same face to the Earth, you have explained that fact, and if you can answer the question why no one can be elected King in Greece, you have given an explanation. But this must be qualified. For although there is the intuition that an explanation is an answer to a why-question, there is also the intuition that not to just *any* answer to a why-question is an explanation (Salmon, 1992). Answers to why-questions that are *not* explanations, include requests for a justification (why is this a fair decision?), requests for evidence (why do you think that the moon always shows the same face to the Earth?), and requests for consolation (why did my partner die?—meant not as a request for medical information).

These, then, are basic and widespread intuitions about explanations. As indicated, it is possible that these intuitions come into conflict with what practitioners of the humanities present as explanations: the theses offered are claimed to be explanations, but they don’t conform to the intuitions about explanation. When that happens, theoreticians about explanation must consider three scenario’s and decide between them. One: keep the intuitions, and reject what is presented as explanations as mere pseudo-explanations or no explanations at all. This resembles what Chisholm has called ‘methodism.’ Two: maintain that what is offered are genuine explanations, and seek to modify or complement the intuitions about explanation—the examples offered should be used to lead the way either to so far unnoticed intuitions about explanation, or to the abandonment of one or more intuitions about explanation. This bears a resemblance to what Chisholm has called ‘particularism’. Three: keep some of the putative examples of humanistic explanation and reject others, and keep some of the intuitions about explanation, and either reject or modify some of the others, or expand the set of intuitions. This is similar to what ethicists have called the ‘reflective equilibrium approach’.

The discussion between proponents of these scenario’s touches on the discussion about explanatory pluralism, the view that there are distinct and irreducible forms of explanation. Explanatory monists argue that there is only one explanatory relation, i.e. only one relation between explanandum and explanans that is truly explanatory. According to some, counterfactual dependence fits that exclusive bill (Baron et al., 2020; Khalifa et al., 2020; Reutlinger, 2016, 2017a; Roski, 2021), according to others it does not (Pincock, 2018, p. 43).

In Sects. 4–6, we showcase theses that are presented as explanations by practitioners of the humanities. We suggest that the proffered explanations are in conformity with the intuitions about explanation, and that they are answers to why-questions of the right kind. Since, as will emerge, these alleged kinds of explanation are very different from each other, our discussion is suggestive of explanatory pluralism. This may be deemed unsatisfactory for the same reason that it is deemed unsatisfactory, even a scandal, that there is no single unifying theory of explanation in the sciences. But if there *can* be no such unifying theory, then we should be glad with having a clear sight of the diverse kinds of genuine explanation that practitioners of the humanities as well as of the sciences offer.^1^

Next, what is a *causal* explanation? It is an explanation that makes an essential reference to a cause, an explanation, that is, where the explanatory relation is causal dependency. Now, there are competing accounts of causation, including counterfactual, process, probabilistic, regularity, and interventionist accounts (Beebee et al., 2009). There are also distinct domains of application of causal language, from physics to sociology to law. To avoid reliance on a single, controversial theory while nonetheless clarifying the notion of causation at least somewhat, we use the so-called ‘Russellian’ criteria that have also been used by others for purposes of distinguishing between causal and non-causal explanations (Reutlinger, 2018):

1. asymmetry: if A causes B, then B does not cause A,
2. time asymmetry: effects never precede their causes in time,
3. distinctness of the causal relata: cause and effect do not stand in a part-whole, supervenience, grounding, determinable–determinate, or any other metaphysical dependence relation,
4. metaphysical contingency: causal relations are metaphysically contingent

These are necessary conditions for a relation to be causal. With respect to explanations, this means that if an explanation is to be causal, the four criteria must be satisfied by the explanatory relation, i.e. the relation between explanandum and explanans. If one of them is not, we can safely conclude that the explanation is non-causal. What are the humanities? If we follow the pragmatic or institutional demarcation (Bod, 2013, p. 2), the humanities include linguistics, musicology, philology, philosophy, theology, literary studies, theatre, film, and media studies, and historical disciplines, including (art) history and archaeology. This demarcation is not, or not entirely, arbitrary. Plausibly, some themes are central to the humanities: they exhibit a focus on methodological principles which are used to search for patterns (see Bod, 2013, p. 5), as do the natural sciences, but not exclusively so. For the humanities deprioritize the universal compared to the particular (think of historiography^2^), accept subjective influences from the researcher’s perspective as legitimate, they aim to not just put forth descriptive claims but also prescriptive ones, and value individual insight independent of scholarly consensus (Foley, 2018). Also, the humanities, generally speaking, study objects that display and express meanings, intentions, and values (van Woudenberg, 2018). The humanities, then, plausibly share at least some fundamental assumptions and features.

As we have indicated, this is an exploratory study, which means we have not looked into all humanistic subdisciplines. We selected four disciplines that are quite different from each other: archaeology, historiography, linguistics, and literary studies. Each of these fields has a significant history and displays an impressive depth and breadth of scholarship.^3^ In the next section, we describe how we selected the types of non-causal explanation in these fields.

## Methodology

This section describes the procedure that we used for selecting proffered explanations.^4^ The methodology applied ensures that the presented explanations aren’t outliers or mere flukes.

### Genre Selection

In order to spot widely accepted humanistic explanations, we selected university-level introductory textbooks and searched for what they explicitly present as explanations. We did this on the assumption that widely used textbooks are seen as providing faithful pictures of a discipline. The authors of textbooks are usually senior researchers with a perceptive eye for theoretical and philosophical debates. A significant number of textbooks, moreover, explicitly address debates about scientific explanation in their fields. In sum, textbooks, we assume, are plausibly the most accessible and representative way to collect widely accepted forms of humanistic explanation.

### Book Selection

We used Google Scholar to cast the net as wide as possible. Due to its citation policies, Google Scholar gives some insight into the popularity of academic textbooks. The search terms we used were “introduction to [name of the discipline]”, in our case: archaeology, linguistics, history and historiography, and literary theory or literature. We used five pages of search results and used the nine titles that had the highest number of citations. The focus on citations was chosen to ensure the representativeness of the result: large amounts of citations indicate that the textbooks are widely regarded as accurate and suitable introductions to the discipline. Papers and non-textbooks were excluded, as were titles from before the year 2000 (although some new editions of older textbooks were included) so that the results consist of contemporary scholarship that is widely seen as appropriate for educational purposes. The selection is biased towards older textbooks, but this is intentional: very recent textbooks have not yet shown whether they are accepted by their respective disciplines. We included companions, as long as they served introductory purposes. For each field, we added one additional title to the nine books which was written by a respected scholar. Although these extra publications did not rack up enough citations, they nonetheless seemed to contain particularly interesting non-causal explanations. The choice for the number of ten books meant that all but one of the forty textbooks had at least a hundred citations, and most had over a thousand. The full book list is added as an Appendix to this paper.

### Search Procedures

The books were searched by tracking the use of the words ‘explain’, ‘explains’, ‘explaining’, ‘explained’, and ‘explanation’. Of course, other semantic markers may also indicate that an explanation is offered, for example words like ‘due to’, ‘because’, ‘in virtue of’, ‘understand’. However, to avoid problematic ambiguities and arbitrary delineations, we searched for explicitly so-called explanations only. This also kept the search process within manageable limits. Also, our inventory only registered (kinds of) explanation that are deemed acceptable by the authors of the textbooks.^5^ Since we focused on *kinds* of explanation rather than on the content of particular explanations, the particular examples we present below need not be true or fully convincing.

### Analysis

The first job was to apply the Russellian criteria. After that, the most theoretically interesting explanations were selected for inclusion and sorted into categories to avoid overlap. For the classification of various explanations, we generally tried to stick to the terminology that is commonly used in the disciplines themselves. However, most explanations were not explicitly classified as being of a certain type. That need not imply that their nature is unclear. Most of the time, the description of the explanans made it clear enough (for example, when the intentions of a person were cited in an action explanation). Sometimes, new or less well-known terms seemed to be necessary to describe the nature of some of the explanations. Those descriptions are tentative. The demarcations between the various categories are based on what seem to be genuine differences in terms of explanatory relations.

### Limitations

As indicated above, this study is exploratory and limited in the five respects we mentioned. To this we add that, as described in 3.3, it is also limited in that we haven’t studied explanations that are not explicitly called ‘explanations’.

## Intentional Explanations

Many explanations that are mentioned throughout our textbooks concern the explanation of human actions and/or their intended results. Many human actions and their outcomes are held to be explainable, or explained, by reference to the intentions or purposes of the actor. In these cases, the explanandum is a human action, and the explanans a goal-directed state of mind of the actor. These states of mind include intentions, aims, goals, desires, reasons, beliefs, and evaluations.

An apt name for explanations of this kind is ‘intentional explanations’, or ‘teleological explanations’, or also ‘personal/agential explanations’. And philosophers have actually used all of these names.^6^ From the textbooks we select two examples and discuss whether they are explanations, and, if so, whether they are non-causal.

Our first example concerns the striking fact that whereas historiography in Germany and other European countries experienced a *Methodenstreit*, historiography in the US did not. What explains this fact? One explanation refers to the intentions and goals of the American ‘New Historians’, who saw historiography as a tool for reshaping society in a progressive spirit. According to Breisach, methodological controversies would only undermine the New Historians’ scientific authority and hamper their political goals, so they chose to refrain from such disputes (Breisach, 2007, p. 290). German and European historians in general, by contrast, did not have anything like the goals that the New Historians had, which is supposed to explain the striking fact.

Our second example, of archaeological provenance, concerns the emergence of state-organized societies. This emergence is explained by reference to the intentional competition for power and resources. More specifically, the explanation talks about “fast-moving diplomatic and economic games where the strongest and most decisive leaders survived” (Fagan & Durrani, 2015, p. 51). This indicates a rational choice or game-theoretic perspective, combined with the implicit assumption that human beings frequently need and desire power and resources, and that once a (violent) competition for resources has started, playing the game is no longer optional if one wants to survive. The specific moves leaders make can be explained as intentional engagement in competition due to felt needs and desires. The competition, plus the realization that an organized state would give a people an advantage in the ‘game’, explains the origin of states and civilization.

Let us now ask: how do these examples fare in light of the intuitions about explanations? To explain, intuitively, is to understand, or to render intelligible. In these two examples, the explanans refers to intentions, goals, choices, and desires. And generally speaking, references to intentions and the like, do tend to render actions and behaviors intelligible—such references, when adequate, give insight into the modal space surrounding the explananda; given the intentions etc. it was, to some degree, to be expected that the explananda would occur. Also, explanation, intuitively, are answers to why-questions of the right kind. The examples offered *are* answers to such questions: “Why was there no *Methodenstreit* among historians in the US?” and “Why did state-organized societies emerge?” Our textbooks clearly indicate that historians and archeologists agree that the two examples on the table are explanations, or attempts thereto. And we can see why they think so: they conform to the intuitions about what explanations are.

Next question: are these putative explanations of the non-causal variety? So, do they meet the Russellian criteria? Beginning with asymmetry: if we suppose that goals or intentions cause the actions of not to engage in a *Methodenstreit*, and the buildup of state-organized societies respectively, we see that in both examples the goals or the actor’s intentions cause the actions, but the actions don’t cause the goals or intentions. Time asymmetry may seem satisfied as well, as in the examples offered the actions precede the attainment of the goals that explain the actions. However, this isn’t quite right, for the explanandum is the action (of attaining some goal), and the explanans the intention (to attain that goal). And if we put it like this, it isn’t so obvious anymore that the intentions precede the actions. There must, of course, be an initial intention that precedes the action in time. But it doesn’t *merely* precede. This comes out when we consider the third criterion: distinctness. If we think that intentions and actions stand in the cause-effect relation, it is also clear that cause and effect are *not* distinct from each other—not in the way that the shining of the sun and the heating of a stone (that is due to the shining) *are* distinct. For the actions are ‘guided’ by the intentions. The intention is a constitutive element of the action. The action would not be the action that it is, if it wasn’t guided by the intention. The action of not engaging in a *Methodenstreit*, and the action of building state-organized societies, are the actions they are due to the fact that they are guided by certain intentions to reach certain goals—the intentions of the actors are constitutive elements of the actions they perform. If we are right about this, this means that the intentional explanation of action does not satisfy the third Russellian criterion, and hence that it is not a causal explanation. To this we add that if we are right, and action and intention are not distinct from each other, it seems wrong to say that the *relation* between action and intention is contingent. If actions have intentions as constitutive elements, the relation between the two is an essential one, and hence the fourth Russellian criterion is not satisfied.

If our discussion is on the right track, then intentional explanations are distinct from causal explanations. And this meshes well with some things that various anti-causalists about action explanation have brought to our attention. They have pointed out, for instance, that intentional states like aims, reasons, evaluations etc. (the ‘causes’ of the actions) have properties that the causes that figure in causal explanations lack—*normative* properties. For example, intentions can be wise or stupid, reasons can be solid or not, emotions can be appropriate or not, and evaluations well or ill-grounded. But there are no fallacious, tenuously supported, or factually wrong causes of the sort that figure in causal explanations (Sehon, 2005, p. 63). Causes just are (cf. Craver, 2014, p. 40; Danks et al., 2014; but, see Hart & Honoré, 1985).

Our textbooks, then, present intentional explanations of human action as legitimate. Of course, Donald Davidson and many who were influenced by him, have aimed to show that teleological explanations can be reduced to causal explanations.^7^ Whether the reduction claim can be made to stick is hotly debated.^8^ This is not the place to discuss this. For here, the point is that in the four established subfields in the humanities, teleological explanations are used and considered to be *bona fide*. And we have suggested that such explanations do not satisfy the Russellian criteria, and hence are non-causal.^9^

But are intentional explanations really explanations? Well, as indicated, they conform to the intuitions about explanation, and they are answers to why-questions of the right kind. So we say: yes they are explanations. But disagreement here is possible; critics might argue that the proffered explanations don’t conform to the intuitions about what an explanation is, that intentions don’t render actions intelligible. Or critics may argue that the why-questions at hand are not of the right kind. We don’t intend to enter that discussion. For now the point is: intentional explanations as offered in the humanities, are, *prima facie*, *bona fide* explanations of a non-causal kind.

## Formal or Structural Explanations

Our textbooks also report explanations that involve abstract structures or forms. Logic, linguistics, and literary studies all know explanations that can be dubbed ‘formal’. As we will see, the kinds of forms or structures involved vary between the disciplines, from logical relations and syntactical structures in linguistics, to plots in literary studies.^10^ This section discusses formal explanations in these disciplines and ends with a discussion of ‘how-possibly?’ explanations.

### Logical Explanations in Linguistics

Linguistics makes extensive use of propositional and predicate logic, so it should come as no surprise that it employs logic in explaining linguistic facts. Take, for example, the following explanandum: Why does “Either Joe is crazy or he is lying, and he is not crazy” entail “Joe is lying”? Here we have a why-question on our hands. It is answered, *explained*, by reference to the underlying logical structure of the sentence, viz. that “whenever a proposition of the form ((p ∨ q) ∧ (¬p)) is true, the proposition q must also be true” (Kroeger, 2018, p. 64). This is, of course, a tautology, but that does not imply, says Kroeger, that we have no explanation here, nor that the explanation is vacuous. The question why one sentence entails another (the explanandum), *is* answered—answered by reference to the underlying logical structure (which is the explanans). Also, the case can be made that an explication of the logical form of the first proposition, ((p ∨ q) ∧ (¬p)), renders it intelligible that the second proposition, q, does indeed follow from it. So what we have matches the intuitions about explanation.

When we check whether the proposed explanation is causal, we can see that it is not. It doesn’t meet the time asymmetry, nor the metaphysical contingency criterion. It doesn’t meet the former as there is no temporal relation between the two sentences on the one hand, and the logical tautology on the other. It doesn’t meet the latter as the relation between the two sentences and the tautology is metaphysically necessary, not contingent. Hence, the proposed explanation, by the criteria we wield, is non-causal. It is natural to call it a ‘formal’ explanation, a general characterization of which will be offered later on.

### Grammatical Explanations in Linguistics

Semantics and syntax are subfields of linguistics that deal, respectively, with word meaning and the structure of linguistic expressions.^11^ As our textbooks indicate, in these subfields we find explicitly so-called explanations.

We start by considering an example of a semantic explanation. It concerns the word pairs ‘man’/‘woman’, ‘stallion’/‘mare’, ‘ram’/‘ewe’, and the relations between these word pairs. The question is: why are the members of each pair in the same way related to each other—so, why are ‘man’ and ‘woman’ related to each other in the same way as ‘stallion’ and ‘mare’, and in the same way as ‘ram’ and ‘ewe’? The answer that is offered to this why-question (so the explanation of the fact that the members of the pairs are similarly related), avails itself of so-called ‘componential analysis’—a linguistic framework that explains word meanings in terms of its ‘semantic parts’. Applied to the question under consideration, this amounts to the following: the meaning of ‘man’ and ‘woman’ derives from its component semantic parts. For example, ‘man’ has as semantic parts {‘human’ and ‘male’}, and ‘woman’ has as semantic parts {‘human’ and ‘female’}; ‘stallion’ has as semantic parts {‘horse’ and ‘male’}, ‘mare’ has {‘horse’ and ‘female’}; ‘ram’ has as semantic parts {‘sheep’ and ‘male’} and ‘ewe’ has {‘sheep’ and ‘female’}. The answer to the why-question is this: the members of each pair have one semantic part in common (‘human’, ‘horse’, and ‘sheep’ respectively) and of each pair one of the members has ‘male’ as a semantic part, and the other ‘female’. The idea, then, is that the meaning relation between ‘man’ and ‘woman’ is explained by their components; and this also explains why the relation between ‘man’ and ‘woman’, between ‘stallion’ and ‘mare’ etc. is the same (Kroeger, 2018, p. 125).

So we have an answer to a why-question. And the componential analysis, it would seem, render the relations within the word pairs intelligible. This suggests that we do have a *bona fide* explanation here.

It is easy to see that the explanation offered is not causal. None of the Russellian criteria are satisfied. It is unclear that asymmetry is satisfied. But it is clear that time asymmetry is not: the explanation doesn’t involve a temporal process. Nor is there distinctness of causal relata: it rather seems as if ‘cause’ and ‘effect’ stand in a part-whole relation, which makes for non-distinctness of the relata. And the relation between ‘man’ and its semantic parts, as well as the commonalities among the relations between the pairs are not contingent. The explanation is thus atemporal and non-causal (Kroeger, 2018: 124). It is a formal explanation (to be characterized later on).

Another semantic phenomenon of which linguists have given an explanation is referential opacity. The sentence “Oedipus wants to marry his mother”, as we all know, is ambiguous. Why? Because Oedipus wants to marry Jocasta, but does not *know* Jocasta is his mother (Croft & Cruse, 2004, p. 35), and if he had known that, he would not have wanted to marry her. The sentence, then, is ambiguous between “Oedipus wants to marry the person who he believes/knows is his mother” and “Oedipus wants to marry the person whom he doesn’t believe/know to be his mother”. One explanation of the ambiguity uses a model that represents the mental ‘belief space’ of a person. In this space, different individuals/items occupy different places. These places may differ from their places in ‘reality space’, i.e. the real world. Jacosta does not fill the role ‘my mother’ in Oedipus’ belief space, but she does in the reality space. Croft and Cruse explain that “many puzzling semantic phenomena are the result of the fact that a value in one space can be described by the role its counterpart has in another space, even if that role is invalid for the value in the first space” (36).

Again, this is an answer to a why-question. And the notion of ‘belief space’ renders it at least to some extent intelligible what is responsible for the referential opacity. Furthermore, by the Russellian criteria, the explanation is not causal. The criterion of time asymmetry is not satisfied. Hence this is another example of a *prima facie bona fide* non-causal explanation. The explanation is formal in a way to be characterized later on.

Syntactical ambiguity is another linguistic phenomenon of which explanations have been given. Why is the sentence “The mother of the boy and the girl will arrive soon” ambiguous? This why-question finds an answer by reference to the syntactical structure of the sentence—a structure that can be described by means of technical terms, *phrase markers*, like ‘noun phrase’, ‘verb phrase’, ‘prepositional phrase’, and so on. The part ‘and the girl’ could either be a separate noun phrase, which would mean the mother and girl are unrelated, or it could be a prepositional phrase, which would mean the mother in the sentence is also the mother of the girl. The authors of the textbook who discuss this example conclude that “whereas an unambiguous sentence is associated with just one basic phrase marker, a structurally ambiguous sentence is associated with more than one basic phrase marker” (Akmajian et al., 2010, p. 181).

Again we have an answer to a why-question. Moreover, the answer, that makes use of the notion of *phrase marker*, renders the ambiguity intelligible. In addition, the relation between explanandum and explanans is not causal, the Russellian criteria are not satisfied. The proffered explanation is non-causal; it is a formal explanation.

Problematic combinations of words are another linguistic phenomenon that merit explanation. Why are sentences like “John drank his sandwich”, “her grandmother swallowed a participle”, and “Susan caramelized her reputation” nonsensical despite containing only meaningful words and complying with all syntactical rules? This why-question is answered by what linguists call ‘selectional restrictions’ (philosophers know them as ‘category mistakes’): many words have implicit restrictions on their domain of application (Kroeger, 2018, p. 121). The use of ‘drank/drinking’ is restricted to beverages, the use of ‘swallow/swallowing’ to edible items, the use of ‘caramelize/caramelization’ to sugars.

Kroeger offers an answer to a why-question, and the answer, that refers to ‘selectional restrictions’, renders the answer intelligible. The proffered explanation is moreover non-causal, as the time asymmetry criterion is not satisfied. The explanation, like all syntactical explanations, are formal.

### Formal Explanations in Literary Studies

Literary scholars do many things. They engage in interpretation, roughly, providing specifications of a text’s meaning (van Woudenberg, 2021, p. 140). But they do more. As our textbooks reveal, at least some literary scholars hold that it is part of their task “to explain why [the text] is as it is” (Eagleton, 2006, p. 92). The task of explaining why a text is as it is could be accomplished by an intentional explanation of the text’s features (see Van Woudenberg, 2021, chapters 6 and 7). However, our textbooks also report explanations of why a literary work is as it is that are of a more formal or structural kind. Such explanations are the focus of this section. We first offer some examples and briefly discuss the relation of such ‘formal’ explanations to intentional explanations.

The background of our discussion is that literary works exemplify structures, an idea that traces back at least to Aristotle’s *Poetics*. Aristotle identified a number of linguistic and literary forms that are used in ancient Greek literature (Klarer, 2004, p. 82), such as metaphors, analogies, symbols, plots, chronology, genre, rhetorical constructions, rhyme, rhythm, feet, etc. The purpose of this section is to illustrate that forms of these kinds figure in literary studies explanations.

Take *plot*. A traditional plot has four parts: exposition, complication or conflict, climax or turning point, and resolution (Klarer, 2004, p. 15). However, not all fiction follows traditional conventions. Kurt Vonnegut’s novel *Slaughterhouse-Five*, for example, mixes different levels of time and action, such as the experiences of a young soldier in World War II, his life in America after the war, and a science fiction-like dreamworld in which the protagonist is kidnapped by an extraterrestrial force. All three levels are juxtaposed as fragments by rendering the different settings as well as their internal action sequences in a non-chronological way. Why is that? Why is the text structured this way? One of the textbooks reports that the absence of a chronological ordering can be explained by the non-linear plot structure, and that the absence of a traditional plot structure can be explained by reference to the fact that Vonnegut’s novel belongs to the genre of the postmodern novel—a genre that makes do with the features of traditional novels (Klarer, 2004: 15–16). Or, to use another example, why does a particular novel contain mostly descriptions and virtually no dialogues? An answer could be that the novel belongs to the genre of naturalism, a hallmark of which is extensive description.

These answers to why-questions do seem to render the explananda at least to some degree intelligible. Moreover, the set of Russellian criteria is not satisfied; it isn’t that genre conventions *cause* the traditional, postmodern or naturalist novel to be what they are. Rather, genre and specimen stand in a determinable/determinate relation. So, literary scholars proffer non-causal explanations of certain features of novels by reference to genre characteristics.

One may wonder whether these examples are ‘really’ explanations. For it seems natural to continue and ask *why* particular genre conventions are or were in place at certain times, and *why* they are or were followed. A full explanation will need to answer these questions as well. Still, the textbooks suggest that the examples mentioned are considered to be explanations by practitioners in the field. A wise response to this worry might therefore be to say that since reference to genre-features does at least to some extent render the explananda intelligible, there exist *bona fide* but partial explanations in this area of scholarship. We should add to this that almost all explanations in the natural sciences are partial too. Newton’s law of universal gravitation explains a wide array of phenomena—but it the law itself is not explained, which means, for example, that the explanation of spring tides by reference to that law, is partial.

How do the formal explanations described above relate to intentional explanations? Can the former be reduced to the latter? There is widespread skepticism about the explanatory value of authorial intentions (see Bertens, 2014, pp. 22–23, 76, 209–212, 155), which, if the skepticism is justified, mars such reduction. But even if such skepticism is misplaced (see Van Woudenberg, 2021, chapters 6–9), this doesn’t entail that formal explanations can be reduced to intentional ones. For the explanantia in the formal explanations in literary studies that we have described do not refer to authorial intentions at all—and do not seem to be reducible to intentions. Intentional and formal explanation of works of literature are entirely compatible. And they are compatible because they target distinct explananda. That said, formal and intentional explanations can figure in the same ‘explanatory chain’, so to speak, since the author may have intentionally adopted the conventions belonging to a particular genre, or the use specific techniques, structures, and other features. And this chain can be continued, for it can be asked *why* certain genre conventions were around in the first place. This brings us back to the partiality of explanations.

The literary studies explanations touched upon in this section, are formal, in a sense to be specified.

### Formal How-Possible? Explanations

Another kind of formal explanation in the textbooks concerns the question how something X is possible, and the answer is that the formal properties of a set of ideas, or of a system of communication like language, necessarily entail that X is possible.

As an example, consider the question asked by historians, feminist theorists, and literary scholars alike: How was the rise of feminist discourse possible in a world that was dominated by patriarchal ideology, with a language that was ‘made by men’ and characterized by sexism (Moi, 2002)? How was it possible that at least some women managed to resist that power and to voice their opposition to it? Moi describes two explanations. The first is that patriarchal ideology is contradictory, “marked by gaps, slides and inconsistencies” (26). Women were supposed to be happy with male dominance, yet they still needed to be controlled by men; they were sometimes considered to be more virtuous than men, yet at the same excluded from positions of power. The contradictory nature of the patriarchal ideology is a logical fact, which holds independently of any causal system. The contradictory nature of the ideology entailed the very possibility of its criticism. In *any* possible world containing such a patriarchal ideology, it is possible to articulate opposition to it, as the ideology contains the seeds of its own critique.

The second explanation that Moi describes is that language is not just a reflection of social relations, but that it is also productive (Moi, 2002, p. 157). The idea is that the syntactical rules allow for combining linguistic expressions in new ways. It is always *possible* to rearrange words so as to create a new sentence with a novel meaning. And *any* kind of language with such basic features opens the possibility for opposition to a dominant but contradictory ideology, since the ideology cannot destroy the logical and linguistic possibility of opposition without destroying language as such.

Are this really explanations? An initial worry might be that what we have here is not an answer to a why-question, but to a *how*-question. Explanations of the sort offered by Moi have been called ‘how-possible? explanations’ (Bokulich, 2014; Dray, 1957; Forber, 2010)). If how-questions cannot be cast as why-questions, we should note that the intuition isn’t that *only* answers to why-questions can qualify as explanations. Moreover, it would seem that the how-question, at least the particular one discussed by Moi, can without too much strain be recast as a why-question: “How was the rise of feminist discourse possible?” isn’t really different from “Why was the rise of feminist discourse possible (in the first place)?” In addition, *a prima vista* it looks that what Moi presents does render the rise of feminist discourse at least somewhat intelligible: a contradictory ideology makes critique possible, as it contains the seeds of its own criticism; the productivity of language makes it possible to formulate criticisms of ideologies. What Moi presents is peeking into the modal space surrounding patriarchism. This suggests that what she presents are putative *bona fide* explanations.

These explanations do not meet the Russellian criteria of temporality and contingency, and hence are not causal explanations. They are, we say again, formal explanations. More on this notion in the next section.

### Formal Explanations Characterized

We repeatedly said that the putative explanations discussed in this section are *formal* or structural explanations. Such explanations are characterized by the fact that the explanatory work is done by (reference to) atemporal complex formal structures. The structures in the examples are (A) Logical structures underlying sentences that express propositions. (B) Semantic structures of words. (C) Belief space structures. (D) Grammatical structures. (E) The structural features of literary genres. (F) Possibility spaces.

That the items just mentioned are *structures* means that they are *ordered*. What this means is that they contain elements, or parts, that are related in orderly ways. It is a task of some magnitude to describe each of these structures, and the parts or elements they contain. But the intuitive idea is that these structures, and the elements ordered within them, exist.

Finally, the structures are *atemporal*. That is why formal explanations are non-causal explanations.

## Normative Explanations

Our textbooks also describe examples of explanations in which evaluative facts or properties seem to do the explanatory work. For example, the French group of historians around the journal *Annales* was highly successful. Why? Part of the answer that is offered, so part of the explanation, is said to be the “sheer intelligence of their writing” and their opposition to “all forms of chauvinism” (Bentley, 2006: 866). ‘Intelligent writing’ and ‘anti-chauvinism’ are evaluative predicates. Another example is from linguistics. The so-called Altaic hypothesis posits a distant genetic relationship between Turkic, Mongolian, and Tungusic languages. This hypothesis is rejected, however. Why? What explains the rejection? It is that there is “unpersuasive evidence” in favor of the hypothesis, and that many of the supposed similarities between the languages can be better explained by reference to factors other than a common origin (Campbell, 2013, p. 359).

What is offered here are answers to why-questions. And the answers do render the explananda intelligible, at least to some extent. The success of the *Annales* historians is made intelligible, if we assume that quality of writing, for example contributes to success (of the work of *Annales* historians, in this case). And the rejection of Altaic hypothesis is at least to some extent rendered intelligible, if we assume that unpersuasive evidence contributes to the rejection of hypotheses based on it (in this case the Altaic hypothesis).

How do these putative explanations score on the Russellian criteria? Let us check this for the rejection of the Altaic hypothesis. The asymmetry criterion is satisfied, for the rejection is caused by the “unpersuasive evidence” (the evaluative fact that is the explanans), but the “unpersuasive evidence” is not caused by the rejection. Explanandum and explanans are asymmetrically related timewise, as the unpersuasive evidence precedes the rejection. The relata are also distinct, for the rejection is distinct from the unpersuasive evidence. And there is also contingency, as there is no necessary relation between unpersuasive evidence and rejection. Hence, normative explanations satisfy the Russellian criteria.^12^ Are they therefore causal explanations? No, for we said that satisfaction of the Russellian criteria is necessary for a causal explanation, not sufficient. Intuitively, it would seem that normative explanations are not causal. So there must be a further condition on causality that is not satisfied. Several candidates present themselves. (1) The relata in the causal relation must be physical/material items, and (2) The relata must be related in a lawlike fashion. All *bona fide* explanations in the natural sciences seem to satisfy these criteria (which suggests that they are genuine conditions for scientific causal explanations); but the explanations in the humanities discussed in this section do not. We won’t pursue this matter any further now, it is future work for a philosophy of the humanities.

## Conclusion

This exploration of four major humanistic disciplines has revealed a variety of putative non-causal explanations: intentional explanations, various forms of formal explanations, and normative explanations. In the humanities, *bona fide* explanantia include purposes, formal properties, and evaluative facts.

One could worry that what are presented in the humanities as explanations are not really deserving of the name. To address this, we have used two widespread intuitive ideas about explanations: that they are answers to why-questions, and that those answers render the phenomenon to be explained at least to some degree intelligible. We have suggested that the proposed explanations discussed in this paper match these intuitions.

We haven’t discussed the question whether the non-causal explanations as we find them in the humanities can be reduced to causal explanation in a systematic way. But our discussion suggests that the prospects for such a reduction don’t look bright.

Nor have we discussed the question whether explanation monism or explanation pluralism is correct in any systematic way. But our discussion suggests that the most popular contemporary monist account, the counterfactual theory of explanation, is unable to account for the explanations that appeal to metaphysically necessary facts or relations, like those used in logical explanations and formal how-possible? explanations.^13^ Our exploration of humanistic explanations thereby reinforces the existing arguments against the counterfactual account (Díez et al., 2013; Khalifa et al., 2020; Lange, 2021), and indirectly bolsters the case for explanatory pluralism. By all indications, we need more than one kind of relation to account for all *bona fide* explanations. Numerous issues deserve further analysis. How should we classify various explanations? Does the variation in formal explanations make them too heterogeneous? How do they relate to mathematical explanations? These are topics that should be on the agenda of the philosophy of the humanities.

The study of humanistic explanations is fascinating and puts the spotlight on the epistemic achievements of an underappreciated part of intellectual inquiry, and can show what they have to offer to the philosophy of explanation in general. Philosophers of explanation have made a place for mathematical explanations; it is time to do the same for humanistic explanations.

## Footnotes

1 This has an analogy in epistemology, where usually three kinds of knowledge are distinguished: propositional knowledge, knowledge by acquaintance, and know-how (see for example Littlejohn & Carter 2021: 262–82). It may be deemed unsatisfactory that there is no unifying theory of knowledge. But if there *can* be no unifying theory, then we should be glad with having a clear sight of the diverse kinds of knowledge we have.

2 The distinction between history as the past and history as an account of the past is not always clear. The most consistent terminology is to distinguish these as ‘history’ and ‘historiography’ respectively (Tucker 2009: 2).

3 We left out our own discipline, philosophy. But this is not because there are no philosophical explanations; for there are such. See Bertrand (2019), Correia and Schnieder (2012), Schaffer (2018).

4 Often we will drop adjectives like ‘proffered’, ‘proposed’, ‘putative’ in connection with ‘explanation’, but they should silently be inserted. As said in the body of the text, our aim is primarily inventory, not vindicative.

5 For example, historian Sian Nicholas rejects a psychoanalytic attempt to explain Margaret Thatcher’s hard-line social policy by appealing to her allegedly abrupt weaning by her distant mother, or the suggestion that her monetarist philosophy developed due to harsh potty training (Nicholas 2004, 131). Given our inventory aims, this restriction wasn’t strictly necessary.

6 See Woodfield (1976), Swinburne (1992), Schueler (2003), Sehon 2005.

7 See Davidson (1980). Reductionists about teleological explanations have often rallied behind the slogan that ‘reasons are causes’.

8 All the authors mentioned in footnote 8 have argued against the reduction claim.

9 Many historians find them central to their discipline, with some going so far as to reject causal analysis altogether (Tucker 2009, p. 106).

10 We note that we should distinguish between structural and structural*ist* explanations. The latter derive from the framework of structuralist literary theory, while the former merely use literary structures as (part of) the explanans. Not all such explanations are dependent on structuralist literary theory.

11 There are deep questions about what, exactly, distinguishes these areas from each other, and what their fundamental nature is. Since the purpose of this paper is to show that in the humanities many explanations are non-causal, and not to indicate to which exact subfield they belong, these matters can be put to one aside.

12 Although we did not find any such explanations in the humanistic textbooks, there is reason to think that normative grounding explanations are also needed in the humanities. For example, the reasonableness of believing in theory T is grounded in various (known) facts about its accuracy, scope, consistency, fruitfulness, simplicity, and so on. Textbook authors frequently provided reasons and arguments for why some theories are worthy of acceptance because of such theoretical virtues. Normative grounding explanations are atemporal and therefore non-causal.

13 Alexander Reutlinger (2017a: 250) recognizes this problem when it comes to some metaphysical and mathematical explanations but does not offer an immediate solution.
